# Institutional Notes and News

**Published:** 1909-09-18

**Authors:** 


### INSTITUTIONAL NOTES AND NEWS.

### LEGACIES TO HOSPITALS.

In the House of Commons last week Mr. Murray Macdonald asked the Secretary to the Treasury whether he was aware that in Ireland all legacies to hospitals and other charities were exempt from legacy duty; and whether, in view of the increasing difficulty of finding money to manage hospitals, the Chancellor of the Exchequer would insert in the Finance Bill a provision extending this exemption to Great Britain. Mr. Hobhouse, replying for Mr. Lloyd George, stated that the latter was aware of the Irish custom, but regretted that he could not see his way to accept the suggestion contained in the latter part of the question.

### THE WEMYSS MEMORIAL HOSPITAL.

The Wemyss Memorial Hospital, given by Lady Eva Wemyss to Wemyss parish, has been formally handed over to the trustees. The hospital contains wards for women and children, operating-room, doctors' and nurses' rooms, caretaker's house, chapel, and mortuary, and has cost about £5,000. Mr. Carlow, chairman of the Fife Coal Company, Ltd., who presided at the opening, said the hospital was to serve a twofold object—the alleviation of suffering and as a memorial to one whose loss they deplored, whose name would be handed down as the greatest benefactor of the parish, because of the ability and energy displayed in developing the villages, in opening new pits, providing facilities for disposing of the produce in new docks, and for travelling on the tramways.

### JOTTINGS.

The new and old buildings of the City Hospital, Aberdeen, are to be installed with electricity, at a cost of £500.

The Grand Lodge of Freemasons has sent £500 to the National Hospital for the Paralysed and Epileptic.

